# Effects of dosimetric inadequacy on local control and toxicities in the patients with T4 nasopharyngeal carcinoma extending into the intracranial space and treated with intensity-modulated radiotherapy plus chemotherapy

**DOI:** 10.1186/s40880-017-0245-0

**Published:** 2017-09-20

**Authors:** Fen Xue, Chao-Su Hu, Xia-Yun He

**Affiliations:** 1 Department of Radiation Oncology, Fudan University Shanghai Cancer Center, Shanghai, 200032 P. R. China; 2 Department of Oncology, Shanghai Medical College, Shanghai, 200032 P. R. China

**Keywords:** Nasopharyngeal carcinoma, Chemotherapy, Intensity-modulated radiotherapy, Local control, Dosimetric inadequacy

## Abstract

**Background:**

To protect neurological tissues, underdosing occurs in most cases of T4 nasopharyngeal carcinoma (NPC) with intracranial extension. In this study, we aimed to evaluate the effect of dosimetric inadequacy on local control and late neurological toxicities for patients treated with intensity-modulated radiotherapy (IMRT) plus chemotherapy.

**Methods:**

We prospectively enrolled patients who had non-metastatic T4 NPC with intracranial extension treated between January 2009 and November 2013. The prescribed dose was 66.0–70.4 Gy to the primary planning target volume (primary gross tumor volume [GTVp; i.e., the nasopharyngeal tumor] + 5.0 mm). Dose–volume histogram parameters were calculated, including minimum point dose (D_min_) and dose to 95% of the target volume (D_95_). All patients received chemotherapy with the cisplatin, 5-fluorouracil, and docetaxel regimen. Survivals were estimated using the Kaplan–Meier method and compared using the log-rank test.

**Results:**

In total, 41 patients were enrolled. The local partial response rate was 87.8% after induction chemotherapy. With a median follow-up of 51 months, 7 patients experienced failure in the nasopharynx; the 3-year local failure-free survival and overall survival rates of the 41 patients were 87.4% and 90.2%, respectively. The actual mean D_min_ to the GTVp was 55.2 Gy (range 48.3–67.3 Gy), and D_95_ was 61.6 Gy (range 52.6–69.0 Gy). All doses received by neurological organs remained well within their dose constraints. No patients developed temporal lobe necrosis or other neurological dysfunctions.

**Conclusions:**

With relative underdosed IMRT plus effective chemotherapy, the patients achieved satisfactory local control with few late toxicities of the central nervous system. Determining the acceptable extent of dosimetric inadequacy requires further exploration.

## Background

Owing to encouraging disease control results and the capacity for delivering a high radiation dose to the target while sparing adjacent organs, intensity-modulated radiotherapy (IMRT) is the standard treatment of nasopharyngeal carcinoma (NPC) [1, 2]. With modern treatment, the prognostic differentiation between T1 disease and T3 disease is narrowing [3]. However, favorable prognosis remains a challenge for patients with T4 disease (characterized according to the American Joint Committee on Cancer [AJCC] staging system [4]), particularly those with intracranial extension. Because the tumor is located near neurological structures, such as the brain stem, temporal lobes, optic chiasm, and optic nerves, inadequate dose coverage of the target volumes often occurs. Ng et al. [5] reported that a total dose higher than 66.5 Gy was tumoricidal. However, other researchers observed that dose escalation increases the occurrence of temporal lobe necrosis [6] and that the delivery of a high radiation dose to even a small volume in the temporal lobe was unsafe [7]. Addressing adequate dose coverage and normal tissue complication probabilities poses an obvious dilemma. If dose constraints for the brain stem, optic chiasm, and optic nerves are prioritized, even the minimum tumoricidal dose to the entire target volume is not always achievable with the maximum tolerated dose of 54 Gy [8].

Given the chemosensitive nature of NPC, the addition of chemotherapy to IMRT showed promising results for disease control and survival. With the advent of docetaxel, additional combinations of chemotherapy regimens are possible. For the treatment of head and neck carcinoma, adding docetaxel to the classic regimen of cisplatin plus 5-fluorouracil is superior to cisplatin plus 5-fluorouracil [9]. Furthermore, Du et al. [10] reported promising outcomes with good compliance and well-tolerated toxicities of induction and adjuvant chemotherapy using cisplatin, 5-fluorouracil, and docetaxel (TPF) for the treatment of locoregionally advanced NPC.

When a more effective chemotherapy regimen is available, we assumed that IMRT, despite some dosimetric inadequacy, may still mitigate the probability of devastating complications and other adverse effects without compromising disease control. In this study, by imposing strict dose constraints for neurological structures, we aimed to explore the effect of dosimetric inadequacy on local control and treatment-induced toxicities for T4 NPC patients with intracranial extension when treating with IMRT plus the TPF regimen.

## Patients and methods

### Ethical permissions and consent

This single-arm study was approved by the Fudan University Shanghai Cancer Center Institutional Review Board (Reference Number 1410140-8). All patients signed informed consent forms before being enrolled in the present study.

### Patients and pretreatment evaluation

Between January 2009 and November 2013, newly diagnosed, non-metastatic, histologically confirmed T4 NPC patients were prospectively recruited at our center. Inclusion criteria were as follows: (1) pathologically confirmed NPC with imaging evidence of intracranial extension (including the extension to the cranial nerves, the cavernous sinus or the parasellar region, or the prepontine region or the posterior cranial fossa); (2) adequate hematologic, hepatic, and renal functions; (3) Karnofsky performance score of 70 or higher; (4) absence of pregnancy or lactation; and (5) absence of previous malignancy or other concomitant malignant disease. Eligible patients were assigned to receive sequential chemoradiotherapy (two cycles of induction chemotherapy + IMRT + two cycles of adjuvant chemotherapy). During analyses, all patients were restaged according to the AJCC 2010 staging classification [4]. Initial evaluation included the following: medical history and physical examination, blood chemistry tests, chest X-ray radiography/computed tomography (CT), abdominal ultrasound/CT, enhanced magnetic resonance imaging (MRI) of the nasopharynx and neck, and nasopharyngoscopy and bone emission CT. Additional investigations were performed only for those patients with suspicious findings. Dental extraction, if deemed necessary, was performed before radiotherapy.

### Imaging protocol

All MRI images were acquired on the same 1.5-T system (Signa Excite HD, General Electric, Milwaukee, WI, USA) with a head and neck coil. The area from the upper border of the orbit to the inferior margin of the sternal end of the clavicle was scanned. Non-enhanced series included T1-weighted fast spin-echo (FSE) images in the axial and sagittal planes [repetition time (TR) 400–500 ms and echo time (TE) 10–15 ms] and T2-weighted FSE images in the axial plane (TR 4000–5000 ms and TE 80–100 ms). T1-weighted fast spoiled gradient echo fat-suppressed axial and coronal sequences (TR 150–250 ms and TE 2–10 ms) were obtained after intravenous injection of gadopentetic acid (Gd-DTPA) at a dose of 0.1 mmol/kg body weight. The thickness/slice gap was 6 mm/1 mm for the axial plane and 3.5 mm/0.5 mm for the sagittal and coronal planes. Images were assessed by a multidisciplinary team of head and neck cancer specialists in our center, which included experienced radiologists and clinicians.

### Radiotherapy and dosimetric parameters

Patients were immobilized in the supine treatment position with thermoplastic masks. Intravenous contrast-enhanced CT using a slice thickness of 5.0 mm was performed for planning. An 85-cm aperture CT (Philips, Amsterdam, The Netherlands) was used for analog positioning, and the CT images were transferred to the Pinnacle^3^ treatment planning system (Philips Medical Systems, Pinnacle v8.0 m, Milpitas, CA, USA) through local area network. For target delineation, images of the T1 sequences with gadolinium-enhanced MRI were fused with the CT simulation images.

The primary gross tumor volume of the nasopharynx (GTVp) and involved lymph nodes (GTVnd) covered all gross tumors found in clinical and imaging examinations. For the GTVp, involved retropharyngeal lymph nodes and the nasopharynx lesions were delineated according to the post-chemotherapy volume. Intracavity lesions were not delineated if they exhibited regression after induction chemotherapy, whereas involved tissues (e.g., the pterygopalatine fossa) were delineated according to the pre-chemotherapy volume of the primary lesion as shown on initial MRI images. Post-chemotherapy volumes of involved neck lymph nodes were used for GTVnd delineation. The clinical target volume (CTV) included the nasopharynx, retropharyngeal lymph node, skull base, entire clivus, pterygoid fossa, parapharyngeal space, entire sphenoid sinus, posterior one-third of the nasal cavity and maxillary sinus, and drainage of the neck (levels II, III, and Va in patients with N0 lesion and levels II–Vb in patients with N1–3 lesions). Critical normal structures, including the brainstem, spinal cord, optic nerves, optic chiasm, lens, eyeballs, temporal lobes, larynx, and parotid glands, were carefully delineated. The prescribed dose was 66.0–70.4 Gy to the primary planning target volume of the nasopharyngeal tumor (PTVp; i.e., GTVp + 5.0 mm) and 66.0 Gy to the planning target volume of involved lymph nodes (PTVnd; i.e., GTVnd + 5.0 mm) in 32 fractions. The PTV60 (high-risk CTV + 5.0 mm) was prescribed 60 Gy in 32 fractions. The PTV54 (low-risk CTV + 5.0 mm) was prescribed 54 Gy in 32 fractions. All patients were irradiated with 1 fraction daily, 5 days per week.

The planning goal was to deliver at least 99% of the prescribed dose to 95% of the GTVp, without exceeding the dose tolerance of the critical neurological organs at risk (OARs). The dose constraints for each normal organ were set according to the Radiation Therapy Oncology Group protocol 0225 [11]. In brief, the ideal maximum point dose (D_max_) was constrained to ≤ 45 Gy for the spinal cord; ≤ 54 Gy for the brainstem, optic chiasma, and optic nerves; and ≤ 60 Gy for the temporal lobes. In exceptional circumstances, the dose constraints could be relaxed to ≤ 50, 60, and 65 Gy, respectively, to 1% volume of the PTV for the above OARs. The dosimetric parameters for each patient were obtained from the dose–volume histogram, including volumes (V), D_max_, dose to 95% of the target volume (D_95_), dose to 50% of the target volume (D_50_), minimum point dose (D_min_), and maximum dose to 1% of the volume (D_1_).

### Chemotherapy

All patients received chemotherapy using the TPF regimen (docetaxel 60 mg/m^2^ intravenous drip on day 1; cisplatin 25 mg/m^2^ per day intravenous drip on days 1–3; and 5-fluorouracil 500 mg/m^2^ per day with a 120-h infusion). One cycle constituted 3 weeks. Induction chemotherapy was designed for two cycles, followed by IMRT 2 weeks later. Four weeks after the completion of radiotherapy, adjuvant chemotherapy was administered.

### Assessment and follow-up

Adverse events related to chemotherapy and radiotherapy were graded according to the National Cancer Institute Common Toxicity Criteria version 3.0 [12] and Criteria of the Radiation Therapy Oncology Group in 1995 [13], respectively. Late neurological toxicities, such as cranial nerve palsy, temporal lobe necrosis, and spinal cord and brain stem injuries, were assessed according to symptoms, physical examinations, and MRI at the time of local failure or the last follow-up. Assessment of tumor response was based on MRI according to the Response Evaluation Criteria for Solid Tumors version 1.1 [14]. Treatment failure, including relapse or progression, was confirmed with nasopharyngoscopy and biopsy or with unambiguous radiologic evidence of progression on MRI. Local failure-free survival (LFFS) was calculated as the time from the date of treatment to the date of first local failure or the last follow-up; regional failure-free survival (RFFS) was calculated as the time from the date of treatment to the date of the first regional failure or the last follow-up; distant failure-free survival (DFFS) was calculated as the time from the date of treatment to the date of the first distant failure or the last follow-up; and overall survival (OS) was calculated as the time from the date of treatment to the date of death or the last follow-up.

Patients were evaluated weekly during radiotherapy. After treatment completion, follow-up occurred every 3 months for the first 2 years, every 6 months for the following 3 years, and annually thereafter. The last follow-up was in October 2016. Each follow-up included medical history, physical examination, and nasopharyngoscopy. Enhanced MRI of the nasopharynx and neck areas was performed every 6–12 months after treatment. Chest X-ray radiography and ultrasonography of the abdomen were conducted annually. Additional tests were conducted when clinically indicated.

### Statistical analysis

SPSS 22.0 software (SPSS Inc., Chicago, IL, USA) was used for statistical analysis. In all, missing time-to-event data (due to loss to follow-up or no event observed at the time of predefined time of analysis) were censored. If patients were lost to follow-up, the time-to-event data were censored at the time of previous recorded follow-up. If no events were observed at the time of predefined time of analysis, the time-to-event data were censored at the time of last follow-up. LFFS, RFFS, DFFS, and OS were determined using Kaplan–Meier analysis, and survival rates between different groups were compared using the log-rank test. *P* values less than 0.05 were considered statistically significant. Survival curves were generated using GraphPad Prism, version 6.0 (GraphPad Software, La Jolla, CA, USA).

## Results

### Patient characteristics

This study included 41 T4 NPC patients with a definite pathologic diagnosis (World Health Organization type II or III). Patient characteristics and treatment details are presented in Table 1. All 41 patients completed two cycles of induction chemotherapy and IMRT. However, 15 (36.6%) patients discontinued the planned adjuvant chemotherapy because of myelosuppression (*n* = 6), physical intolerance (*n* = 3), or personal reasons (*n* = 6).

**Table 1:** Characteristics of T4 NPC patients with intracranial extension who were treated with IMRT plus chemotherapy

Characteristic	No. of patients (%)
Age (years)	
Median (range)	46 (21–65)
Gender	
Male	33 (80.5)
Female	8 (19.5)
Histology	
WHO type II	1 (2.4)
WHO type III	40 (97.6)
KPS	
70	5 (12.2)
80	27 (65.8)
90	9 (22.0)
N category	
N0	2 (4.9)
N1	23 (56.1)
N2	11 (26.8)
N3	5 (12.2)
Stage	
IVA	36 (87.8)
IVB	5 (12.2)
Completion of chemotherapy	
Induction (2 cycles)	41 (100)
Adjuvant (2 cycles)	26 (63.4)
Adjuvant (1 cycle)	7 (17.1)
Adjuvant (0 cycles)	8 (19.5)

*NPC* nasopharyngeal carcinoma, *IMRT* intensity-modulated radiotherapy, *WHO* World Health Organization, *KPS* Karnofsky performance status

### Dosimetric data

**Fig. 1:**
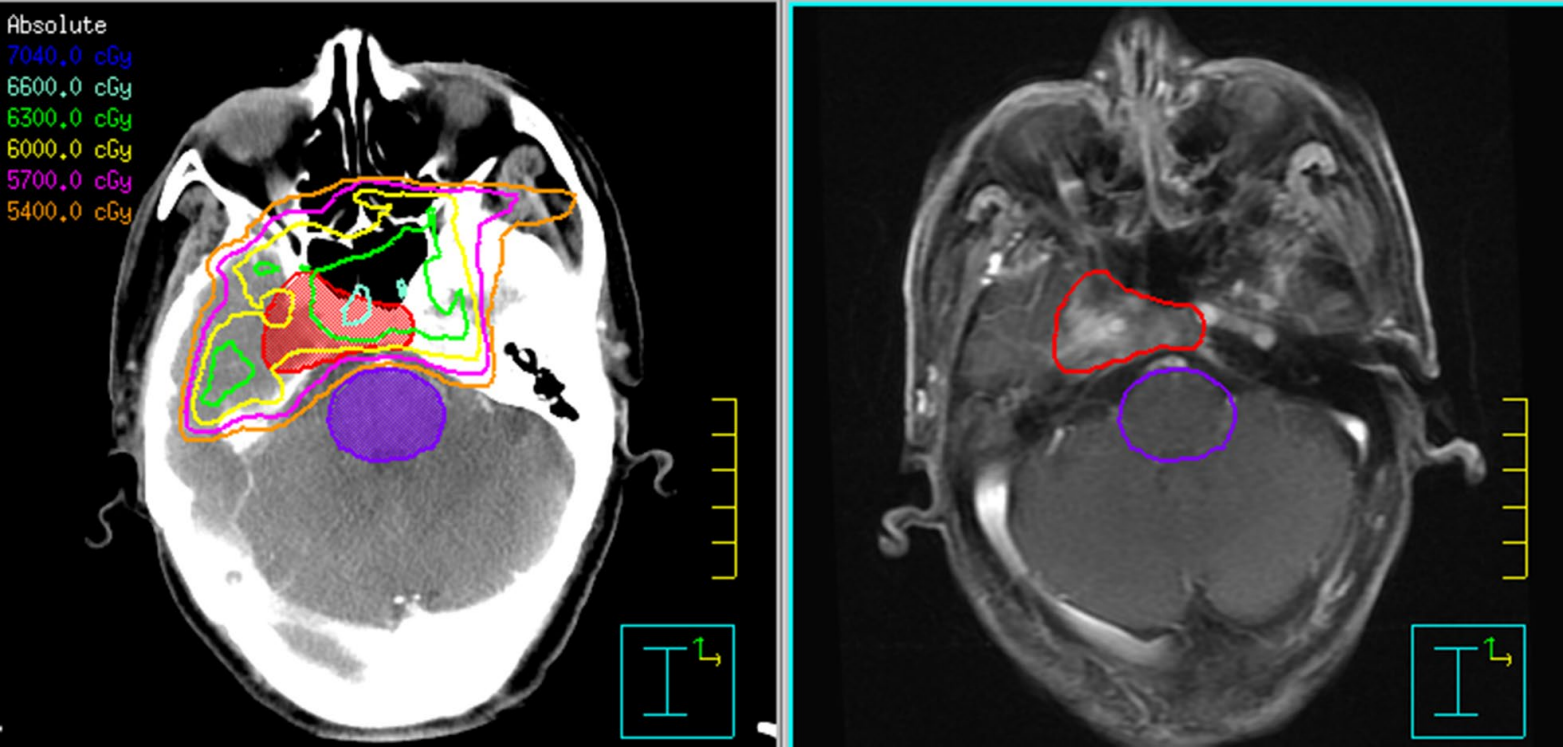
Isodose distribution in a patient with nasopharyngeal carcinoma (NPC) extending to the right cavernous sinus. The right temporal lobe is close to the tumor. The gross tumor volume is delineated with a red line. Most of the tumor was underdosed. Although the maximum point dose of the right temporal lobe was 66.0 Gy, 95% of it received less than 60 Gy. The eyes, brain stem, left temporal lobe, optic chiasm, and optic nerves were spared from high-dose radiation

Dosimetric data for critical neurological OARs and GTVp are summarized in Tables 2 and 3, respectively. Dose constraints of critical neurological OARs, such as the spinal cord, brainstem, optic chiasm, and optic nerves, were strict (Fig. 1). Because of their closer proximity to the primary tumor, affected sides were always covered by a higher dose than unaffected sides. Except in 3 patients who had a D_1_ of the affected temporal lobes that slightly exceeded the criteria of 65 Gy (65.8, 65.3, and 65.2 Gy maximum doses to 1% of the volume), doses to all sides of critical neurological OARs were maintained within their dose constraints. Owing to the tumors’ proximity to critical neurological OARs, all patients received inadequate radiation doses.

**Table 2:** Dosimetric data for neurological organs at risk

Organ at risk	D_max_ (Gy)	D_1_ (Gy)	V_45 Gy_ (%)	V_54 Gy_ (%)	V_57 Gy_ (%)	V_60 Gy_ (%)	V_65Gy_ (%)
Brainstem	55.9 (53.3–58.2)	53.3 (50.8–56.2)	/	1.1 (0–10.9)	0	0	0
Spinal cord	43.9 (41.0–45.7)	41.1 (36.9–44.0)	0	0	0	0	0
Optic chiasm	56.4 (43.1–59.6)	56.0 (41.9–59.5)	/	43.1 (0–98.8)	2.3 (0–47.8)	0	0
Optic nerve							
(A)	54.6 (42.4–60.1)	54.1 (41.9–59.5)	/	14.1 (0–51.9)	1.2 (0–17.3)	0	0
(N)	54.5 (42.4–58.9)	53.9 (42.0–58.3)	/	9.9 (0–44.3)	0.1 (0–3.0)	0	0
Temporal lobe							
(A)	65.1 (60.4–68.4)	63.0 (58.3–65.8)	/	/	/	7.9 (0.1–49.9)	0.2 (0–1.9)
(N)	64.7 (58.8–67.6)	61.9 (53.4–64.5)	/	/	/	4.2 (0–13.2)	0

All values are presented as mean followed by range in parentheses

*D*_*max*_ maximum point dose, *D*_*1*_ maximum dose to 1% of the volume, *V*_*x Gy*_ percentage volume receiving a dose of x Gy or more, (x = 45, 54, 57, 60, and 65), / not applicable, *A* affected side, *N* unaffected side

**Table 3:** Dosimetric data for GTVp

Item	Dosimetric data
GTVp (cm^3^)	64.8 (22.7–166.9)
D_min_ (Gy)	55.2 (48.3–67.3)
D_50_ (Gy)	71.7 (67.7–75.0)
D_95_ (Gy)	61.6 (52.6–69.0)
D_max_ (Gy)	77.1 (75.3–79.4)

All values are presented as mean followed by range in parentheses

*GTVp* primary gross target volume (nasopharyngeal tumor), *D*_*max*_ maximum point dose, *D*_*min*_ minimum point dose, *D*_*50*_ dose to 50% of the target volume, *D*_*95*_ dose to 95% of the target volume

### Local response to treatments

The response to treatments of the nasopharyngeal lesions was assessed based on MRI scans. After two cycles of induction chemotherapy, 36 (87.8%) patients had local partial response (PR), and 5 (12.2%) had local stable disease (SD). By the end of radiotherapy, 25 (61.0%) patients had local complete response (CR), and 16 (39.0%) had local PR. The overall response (CR plus PR) rate for local lesions was 100%. After adjuvant chemotherapy, 10 of 16 patients with local PR had local CR. Twelve months after the completion of treatment, 39 (95.1%) patients had local CR, and 2 (4.9%) still had local PR. The 2 local PR patients eventually developed disease progression (PD) 23 and 30 months after IMRT.

### Outcome analyses

**Fig. 2:**
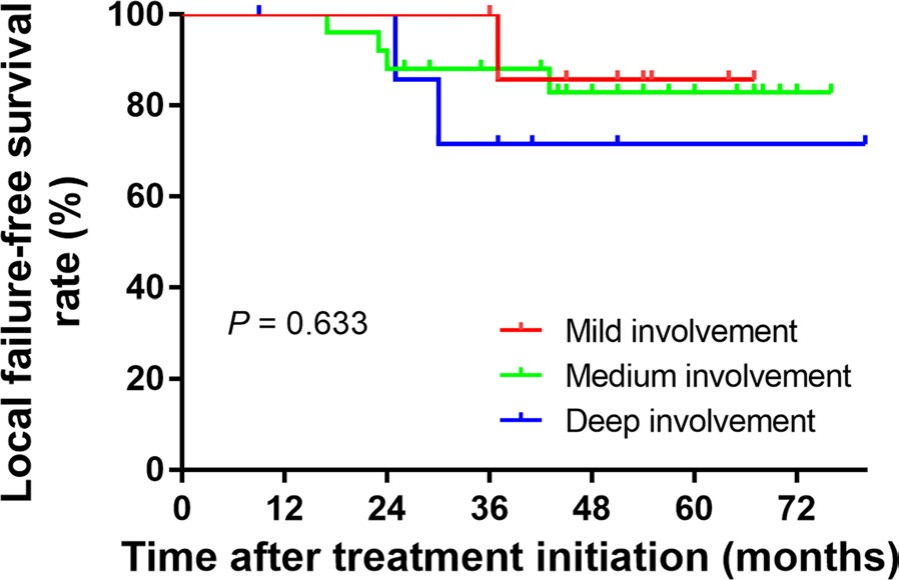
Effect of different intracranial extension levels on local failure-free survival. Kaplan–Meier curves compared with log-rank test showed no significant differences in terms of local failure-free survival among T4 nasopharyngeal carcinoma patients with mild, medium, and deep involvement

With a median follow-up of 51 months (range 9–84 months), treatment failure was observed in 15 patients. Seven patients developed local failure: 5 recurrences and 2 PDs. The only 2 regional failures occurred at 46 and 48 months after IMRT. Distant metastasis occurred in 8 patients (2 combined with locoregional failure), including bone metastasis in 2 patients, liver metastasis in 2 patients, lung metastasis in 1 patient, and multiple metastases in 3 patients. By the last follow-up, 3 patients had died of local failure, 6 had died of distant metastasis, 1 had died of local failure plus distant metastasis, and 2 had died of unknown causes. For all patients, 3-year LFFS, RFFS, DFFS, and OS rates were 87.4%, 100.0%, 89.9%, and 90.2%, respectively. Based on a previous study [15], mild involvement was defined as extension of the tumor to the cranial nerves only; medium involvement was defined as extension of the tumor to the unilateral cavernous sinus or parasellar region only; and deep involvement was defined as extension of the tumor to the bilateral cavernous sinus or the parasellar region, the prepontine region or the posterior cranial fossa. According to these criteria, 8, 25, and 8 patients were classified as having mild, medium, and deep involvement, with 3-year LFFS rates of 100.0%, 88.0%, and 71.4%, respectively (*P* = 0.633; Fig. 2).

### Treatment-related toxicities

Acute and late toxicities by grade are detailed in Table 4. In patients treated with TPF, leukopenia was the most common adverse effect. After induction chemotherapy, the occurrence rate of grade 3 acute leukopenia was 14.6% (6/41). Only 1 patient (2.4%) had grade 4 leukopenia. Grades 3 and 4 leukopenia occurred in 9 (27.2%) and 5 (15.2%) patients who received adjuvant chemotherapy, respectively. No other grade 3–4 acute hematologic toxicities were observed in any patient during chemotherapy.

**Table 4:** Frequencies of chemotherapy-related acute toxicities among the patients

Toxicity	Grade 0, *n* (%)	Grade 1, *n* (%)	Grade 2, *n* (%)	Grade 3, *n* (%)	Grade 4, *n* (%)
Induction chemotherapy (*n* = 41)					
Leukopenia	12 (29.3)	7 (17.1)	15 (36.6)	6 (14.6)	1 (2.4)
Anemia	38 (92.7)	2 (4.9)	1 (2.4)	0	0
Thrombocytopenia	37 (90.2)	2 (4.9)	2 (4.9)	0	0
Liver dysfunction	41 (100)	0	0	0	0
Renal dysfunction	41 (100)	0	0	0	0
Adjuvant chemotherapy (*n* = 33)					
Leukopenia	9 (27.3)	5 (15.2)	5 (15.2)	9 (27.3)	5 (15.2)
Anemia	29 (87.9)	4 (12.1)	0	0	0
Thrombocytopenia	31 (93.9)	2 (6.1)	0	0	0
Liver dysfunction	33 (100)	0	0	0	0
Renal dysfunction	32 (97.0)	1 (3.0)	0	0	0

No radiotherapy-related late neurological toxicities, such as cranial nerve palsy, temporal lobe necrosis, brain stem injury, or spinal cord injury, were observed

The most common radiation-related acute toxicities were grade 1 or 2. Grade 3 acute mucositis occurred in 12 (29.3%) patients, but no patients had grade 4 toxicities. No patients required radiotherapy interruption or termination because of acute toxicity, except for 1 patient who chose to discontinue the treatment for personal reasons after 29 fractions of irradiation. No late neurological dysfunctions, such as temporal lobe necrosis, brain stem injury, or spinal cord injury, were observed.

## Discussion

In the present study, we explored the efficacy and toxicities of IMRT with strict OAR dose constraints plus TPF chemotherapy for T4 NPC patients with intracranial extension. Though dosimetric inadequacy occurred in D_min_, the results showed a satisfactory outcome in terms of local response rate and 3-year LFFS rate, and showed low rates of treatment discontinuation, as well as few late toxicities of the central nervous system. Our results further suggested no significant differences for LFFS among patients with mild, medium, or deep involvement *(P* = 0.633), though those with deep involvement tended to have worse local control.

In the present study, the mean D_min_ to the GTVp was 55.2 Gy (range 48.3–67.3 Gy), which is lower than the 58.9 Gy (range 29.4–72.1 Gy) reported by Cao et al. [16]; however, we reported a 3-year LFFS rate of 87.4%, which is higher than 82.1% reported by Cao et al. [16]. In addition, no neurological dysfunction was observed in our study. This indicated that the dosimetric inadequacy caused by inadequate GTVp coverage might minimize toxicities without compromising local control. In consistent with our results, Lin et al. [17] reported similar 4-year LFFS rates for NPC patients treated with large-target-volume IMRT and reduced-target-volume IMRT, but late toxicities of the central nervous system were less in the latter group *(P* < 0.001). In the NPC-2003-GPOH/DCOG study, children and young adults with stage III/IV NPC received chemoradiotherapy and interferon-beta [18]. During a median follow-up of 30 months, only 6.8% (3/44) of patients with a T4N2 tumor experienced treatment failure. For all patients, the prescribed dose was 54.0–59.4 Gy [18]. It further indicated that, for T4 NPC patients with intracranial extension, treatment with IMRT at dosimetric inadequacy to some extent plus TPF chemotherapy may be feasible.

In locally advanced NPC, survival benefits were observed when chemotherapy was added to IMRT [19]. In recent years, induction chemotherapy has been used in clinical practice with potential advantages in terms of shrinking the primary tumor, which, for patients with intracranial invasion, results in wider margins to normal tissues [20]. As reported in a multicenter, phase III randomized controlled trial (NCT01245959), the addition of induction chemotherapy with the TPF regimen to concurrent chemoradiotherapy improved failure-free survival with acceptable acute toxicity [21]. Moreover, in a meta-analysis, adjuvant chemotherapy was shown to confer the highest survival benefit [22]. In the present study, the TPF regimen, given its superiority in the treatment of head and neck squamous cell carcinoma, was administered in both induction and adjuvant chemotherapy. Sun et al. [23] contended that the advantages of IMRT may “counteract” the effect of concurrent chemoradiotherapy in improving the local control rate. Therefore, according to our previous experience [10], we chose not to administer concurrent chemotherapy to reduce radiation-induced acute toxicities.

Despite the advantages of IMRT, late toxicities of the central nervous system remain the treatment bottleneck for T4 NPC cases [24]. As reported by Su et al. [25], for NPC patients treated with IMRT, temporal lobe injury was not observed in patients with T1-2 disease, but its occurrence rate increased significantly in patients with T4 disease (13.4%) compared with those with T3 disease (3.1%). Higher doses to OARs might account for more late toxicities. Su et al. [25] also found a 2.5% augment of temporal lobe injury per Gy of D_1_ exceeding 52 Gy. Uy et al. [26] reported 1 case of radiation-related brainstem necrosis in 40 meningioma patients with D_max_ to the brainstem of 55.6 Gy; the necrotic area was in the high-dose coverage part of the brainstem, which may also have contributed to the one patient’s death. Therefore, one should be cautious about increasing dose constraints of the nerve system. A previous study from our institution assessed late toxicities in 80 T4 NPC patients and identified 6 cases of temporal lobe necrosis [27]. In this previous study, the mean D_max_ delivered to the affected side of the temporal lobe was 75.9 Gy, and most irradiation hot spots were found in the enhanced region of necrosis [27]. This suggests that, after IMRT, irradiation hot spots may have a close association with late injuries of neurological tissues. Based on the above results, we applied strict dose constraint criteria for D_max_. We observed a D_max_ of 68.4 Gy to the temporal lobes with no temporal lobe necrosis. In addition, the D_max_ to the spinal cord inferior of 45.0 Gy may have contributed to no spinal cord injuries in our present patients, except 1 patient with a D_max_ of 45.7 Gy. No other neurological dysfunctions, e.g., brain stem injury and cranial nerve palsy, occurred in our present study, which we attribute to the strict dose constraints for neurological structures.

Ng et al. [5] found that the minimum dose to the target volumes was related to locoregional failure, and a dose of more than 66.5 Gy was reported to be tumoricidal. They further reported that most T4 diseases (and some T3 diseases) were underdosed (< 66.5 Gy) and that an underdosed GTVp of 3.4 cm^3^ was a prognostic factor for short LFFS [5]. However, adequate dose coverage of target volumes may lead to an unavoidably high dose of irradiation to neurological structures, causing serious late toxicities [8]. In patients with locally advanced NPC, He et al. [28] studied the distance between the primary tumor and the brainstem. For patients with distances > 4.7 versus ≤ 4.7 mm, with a mean D_min_ to the target volume of 63.1 Gy (range 21.7–74 Gy) versus 46.4 Gy (range 21.9–66.6 Gy), respectively, the corresponding mean D_max_ to the brainstem was 51.8 Gy (range 41.7–68.8 Gy) versus 54.1 Gy (range 45.1–68.4 Gy). In patients with locally advanced NPC, the proximity to neurological structures influences the radiation dose that reaches OARs and the tumor. For patients with T4 lesions (intracranial extension), this phenomenon is more common. In fact, one important factor affecting the degree of target dose coverage is the treatment planning protocol. We achieved strict dose constraints to neurological structures with maximum D_max_ of 45.7, 58.2, 68.4, 59.6, and 60.1 Gy to the spinal cord, brainstem, temporal lobes, optic chiasm, and optic nerves, respectively, and 0.2% and 0.04% of V_65 Gy_ (percentage volume receiving a dose of ≥ 65 Gy) to the affected and unaffected temporal lobes, respectively. The mean D_min_ to the GTVp was 55.2 Gy. For some series that used a less strict OAR dose constraint set, higher dose coverage of target volumes was reported. Cao et al. [16] reported a mean D_min_ to the GTV of 58.9 Gy. However, in their series, the maximum point doses to the spinal cord, brainstem, temporal lobes, optic chiasm, and optic nerves were 50.5, 80.3, 87.2, 89.9, and 87.6 Gy, respectively. Optimal dose coverage cannot be obtained without exceeding dose constraints for neurological structures. However, combined with effective chemotherapy, the acceptable extent of dosimetric inadequacy may be redefined with further investigation.

The limitations of this study include the relatively small sample size and short follow-up. Additionally, some injuries to the central nervous system may be asymptomatic or subtle and may not be diagnosed by clinical symptoms and physical/image examination, which may result in low occurrence rates of late injures to neurological structures. A more accurate and careful neurological examination is therefore required. For some cases, the follow-up time was less than 4 years, whereas, the peak time of temporal lobe necrosis was about 3–4 years after radiotherapy. Finally, as a single-arm study, there was no well-matched control group to be compared with. Thus, our results should be considered preliminary and require further confirmation.

## Conclusions

To not exceed dose constraint criteria for neurological tissues, most patients who have T4 NPC with intracranial extension are underdosed. In treatment that comprised IMRT plus effective chemotherapy, we observed that slight dosimetric inadequacy led to satisfactory local control rate with few late toxicities of the central nervous system. However, the acceptable extent of dosimetric inadequacy remains unknown and requires further investigation.
